# Data-Driven Taxonomy for Antipsychotic Medication: A New Classification System

**DOI:** 10.1016/j.biopsych.2023.04.004

**Published:** 2023-10-01

**Authors:** Robert A. McCutcheon, Paul J. Harrison, Oliver D. Howes, Philip K. McGuire, David M. Taylor, Toby Pillinger

**Affiliations:** a Department of Psychiatry, University of Oxford, Oxford, United Kingdom; b Department of Health, Oxford Health National Health Service Foundation Trust, Oxford, United Kingdom; c Department of Psychosis Studies, Institute of Psychiatry, Psychology and Neuroscience, London, United Kingdom; d South London and Maudsley NHS Foundation Trust, London, United Kingdom; e H. Lundbeck A/S, København, Denmark; f National Institute for Health and Care Research Oxford Health Biomedical Research Centre, Oxford, United Kingdom

**Keywords:** Atypical, Category, Generation, Nomenclature, Pharmacology, Psychiatry

## Abstract

**Background:**

Globally, there are more than 25 licensed antipsychotic medications. Antipsychotics are commonly described as either typical or atypical, but this dichotomous classification does not reflect the diversity of their pharmacological and clinical profiles. There is a need for a data-driven antipsychotic classification scheme suitable for clinicians and researchers that maps onto both pharmacological and clinical effects. Receptor affinity provides one starting point for such a scheme.

**Methods:**

We analyzed affinities of 27 antipsychotics for 42 receptors from 3325 in vitro receptor binding studies. We used a clustering algorithm to group antipsychotics based on receptor affinity. Using a machine learning model, we examined the ability of this grouping to predict antipsychotic-induced clinical effects quantified according to an umbrella review of clinical trial and treatment guideline data.

**Results:**

Clustering resulted in 4 groups of antipsychotics. The predominant receptor affinity and clinical effect “fingerprints” of these 4 groups were defined as follows: group 1, muscarinic (M_2_–M_5_) receptor antagonism (cholinergic and metabolic side effects); group 2, dopamine (D_2_) partial agonism and adrenergic antagonism (overall low side-effect burden); group 3, serotonergic and dopaminergic antagonism (overall moderate side-effect burden); and group 4, dopaminergic antagonism (extrapyramidal side effects and hyperprolactinemia). Groups 1 and 4 were more efficacious than groups 2 and 3. The classification was shown to predict out-of-sample clinical effects of individual drugs.

**Conclusions:**

A receptor affinity–based grouping not only reflects compound pharmacology but also detects meaningful clinical differences. This approach has the potential to benefit both patients and researchers by guiding treatment and informing drug development.

SEE COMMENTARY ON PAGE 524

Psychotropic agents have traditionally been classified based on clinical indication (1). In the case of antipsychotics, the drugs used to treat schizophrenia and related psychoses, this classification has also mapped onto a shared pharmacological mechanism of dopamine D_2_ receptor antagonism, which is tightly linked to clinical efficacy (2, 3, 4).

Despite sharing a common dopaminergic mechanism of action, there are significant differences between antipsychotic agents in terms of their broader pharmacological and clinical effects, both in terms of efficacy and tolerability (5, 6, 7). Early attempts to provide a more granular classification of antipsychotics have used an atypical/typical or (almost identical) first-/second-generation dichotomy (8). While initially proposed to reflect mechanistic differences, it has subsequently become clear that the compounds within these categories shared neither common pharmacological nor clinical profiles (9). The subsequent development of a neuroscience-based nomenclature (NbN) was motivated, in part, to address this shortcoming (9,10). The NbN approach categorizes compounds by clinical indication and a summarized receptor profile. However, this process relies, to some extent, on expert judgment and involves a simplification of the highly diverse pharmacology of this group of compounds. Although simplification may be necessary when developing a system that can be applied across the pharmacopoeia, it has the potential to obscure important similarities and differences between drugs.

There are large interindividual differences in antipsychotic response, and many patients switch antipsychotics multiple times before finding one that is both well tolerated and effective (11, 12, 13). There is currently no grouping of antipsychotics to help guide clinicians and patients in their choice of initial or subsequent drug. For patients whose psychotic symptoms have not improved adequately with first-line treatment or who are experiencing side effects, clinical guidelines recommend switching to a different antipsychotic but give little guidance on which drug to select (14,15). Even when there is guidance, it is typically limited to switching between atypical/typical agents, which does not clearly reflect pharmacological profile or clinical effects (i.e., efficacy and side-effect profiles) (14). Thus, a classification system that facilitates a switch to a second-line agent with a distinct pharmacological mechanism of action may improve chances of treatment response and/or tolerability. Recognizing that antipsychotics with similar receptor binding profiles share similar clinical effect profiles may also help drug development. For example, dopamine receptor partial agonists have been heralded for their relatively more benign metabolic side-effect profiles (16); however, in a recent network meta-analysis ranking antipsychotics based on their associated metabolic side effects, ziprasidone and its structural analog lurasidone (both dopamine receptor antagonists) were grouped with the partial agonists as superior agents (5). Comprehensively understanding patterns of pharmacological similarity across compounds may support initiatives to develop safer and more tolerable treatments.

A systematic synthesis of the pharmacology of antipsychotic medication is made possible by the availability of a high number of receptor binding studies covering a wide range of receptor types (17). These studies enable the construction of a receptor “fingerprint” for individual antipsychotics. In this article, we synthesize the results of all relevant receptor binding studies to derive a receptor fingerprint for each antipsychotic. We then apply an unbiased clustering algorithm to group antipsychotics with similar profiles before developing a machine learning model that uses receptor profiles to predict clinical effects. We find that receptor profile–defined groupings show limited overlap with existing classification schemes and map well to clinical effects.

## Methods and Materials

### Overview

We performed a comprehensive search for antipsychotic receptor affinities. We then clustered antipsychotics based on the similarity of their receptor profiles. We next characterized these receptor-defined clusters in terms of their receptor affinities and clinical profiles. Finally, we compared the ability of these clusters to predict clinical effects and compared this with existing methods of categorizing antipsychotics.

### Determining Receptor Affinities

As in previous work (18), receptor affinities for all antipsychotics were obtained if both receptor binding affinities [from the National Institute of Mental Health Psychoactive Drug Screening Program database, https://pdsp.unc.edu/databases/kiDownload/ (17)] and clinical effects [reported in a recent meta-analysis (6)] were available. Only data from studies that reported binding to human tissue were included. A receptor was included in the analysis if data were available for at least 5 separate drugs. Antipsychotic drugs were included in the subsequent analysis if data were available for at least 5 separate receptors. In both cases, the criterion of a minimum of 5 data points was used to avoid scenarios in which drugs or receptors with minimal data could have undue influence upon subsequent analyses. Receptors were removed if Ki values were identical for all drugs because, in this scenario, the receptor Ki values supply no useful information for subsequent analyses. If multiple studies existed for the same receptor and drug, then the median value was calculated and used in subsequent analyses. The median was used instead of the mean due to its greater robustness against the influence of outliers. Finally, Ki values were converted to pKi values as is routine for pharmacological studies.

### Clustering Antipsychotics Based on Receptor Affinities

The minimum pKi value was 4. This value (4) was subtracted from all pKi values to give a floor score of 0. Next, in case that a drug was an agonist or partial agonist at a given receptor [as reported in earlier reviews (7)], the pKi value for that drug-receptor combination was multiplied by −1 to account for the functionally inverse effect. Without this inversion, there would be no distinction between agonists and antagonists.

Probabilistic principal component analysis (PPCA) was then used to impute any missing pKi values (19). Then, the adjusted pKi values for all antipsychotics were Pearson correlated with one another to produce a correlation matrix. In this correlation matrix, a high correlation coefficient between 2 antipsychotics indicates that they share a similar receptor profile. This approach has a similar effect to normalizing for D_2_ pKi (18), thereby accounting for the dosing differences between antipsychotics. The Louvain clustering algorithm was then used to group antipsychotics with similar receptor profiles into distinct groups (20).

### Characterizing the Relationship Between Receptor Profiles, Categorization Schemes, and Clinical Effects

To characterize the receptor profile of the antipsychotic clusters identified above, we performed a PPCA of the receptor profiles, then calculated the mean component loading for the 3 components explaining the greatest proportion of variance for each cluster.

Relative side-effect burden (magnitude or relative risk) for 13 common adverse effects (weight gain, Parkinsonism, akathisia, anticholinergic effects, sedation, hyperprolactinemia, corrected QT interval prolongation, orthostatic hypotension, dystonia, tardive dyskinesia, seizure, dyslipidemia, and dysglycemia) and efficacy (in terms of positive, negative, and total symptoms) of antipsychotics included in the PPCA were obtained from an umbrella review of network meta-analyses and clinical guidelines for the acute treatment of schizophrenia (see the Supplement). For each clinical measure, we characterized the mean for each of the 4 receptor-defined clusters.

We next examined whether complete receptor binding profiles and receptor profile–based groupings (clusters) were predictive of clinical profiles and compared them with existing classification schemes. We developed a prediction model using training data consisting of all but one of the available antipsychotics. Within the training data, any missing clinical effect values were imputed using PPCA. In this model, either the receptor profiles (number of predictor variables, *D* = 42), the receptor profile–defined groupings (*D* = 4), NbN-defined groupings (*D* = 7), or a typical/atypical/partial agonist grouping (*D* = 3) were used as the predictor variables, while the side effect and efficacy scores (k = 16) were used as the target variables. The NbN groupings were defined on the basis of their mode of action as reported at https://nbn2r.com/authors. A definitive typical/atypical distinction is not available, with most drugs (other than clozapine) classified according to the year of discovery.

We used partial least squares regression, given this is a model well suited to using multiple features to simultaneously predict multiple targets. We then used the partial least square model that had been fitted on the training data to predict clinical effects for the single antipsychotic not included in the training data. We then calculated the median of the absolute error between predicted and observed clinical effect scores for this antipsychotic. We repeated this across all 27 antipsychotics and calculated the median error score across the 27 antipsychotics to provide a summary estimate of predictive ability for each of the 4 methods of categorization. We used permutation testing to assess statistical significance of the prediction by comparing the observed median error score with a null distribution of median error scores generated by shuffling antipsychotics in the training data 500 times to break the connection between receptor and clinical effect profile. Analyses were conducted using the python programming language, with the NumPy package used for most of the linear algebra, scikit-learn for the partial least squares prediction modeling, and seaborn for figure generation (21, 22, 23). Code and data for all analyses are available at https://github.com/rob-mccutcheon/antipsychotic_pca_paper.

## Results

### Receptor Affinities

**Figure 1:**
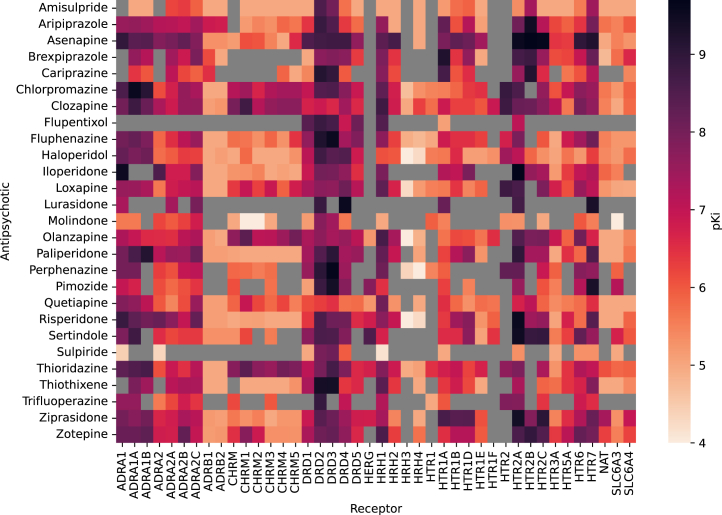
Antipsychotic pKi values. Larger pKi values indicate greater affinity of the drug to the receptor. For visualization purposes, data here represent pKi values with no adjustments made based on whether a drug is an agonist or antagonist, whereas subsequent analyses make this adjustment. Gray squares indicate an absence of data. ADRA, alpha-adrenergic receptor; ADRB, beta-adrenergic receptor; CHRM, muscarinic acetylcholine receptor; DR, dopamine receptor; HERG, human ether-a-go-go-related gene; HR, histamine receptor; HTR, serotonin receptor; NAT, noradrenaline transporter; SL6A3, dopamine transporter; SL6A4, serotonin transporter; SLC6, solute carrier family 6 transporter.

In total, 97,599 Ki values were extracted. Of these, 5304 were related to antipsychotics, of which 3325 reported on binding to human tissue. Data regarding 67 distinct receptors and 29 different antipsychotics were reported, but this was reduced to 42 receptors and 27 antipsychotics when the requirement for ≥5 datapoints for each receptor and drug were applied (2 drugs and 13 receptors were removed) (see the Supplement for details). The pKi values are displayed in Figure 1.

### Clustering Antipsychotics Based on Receptor Affinities

**Figure 2:**
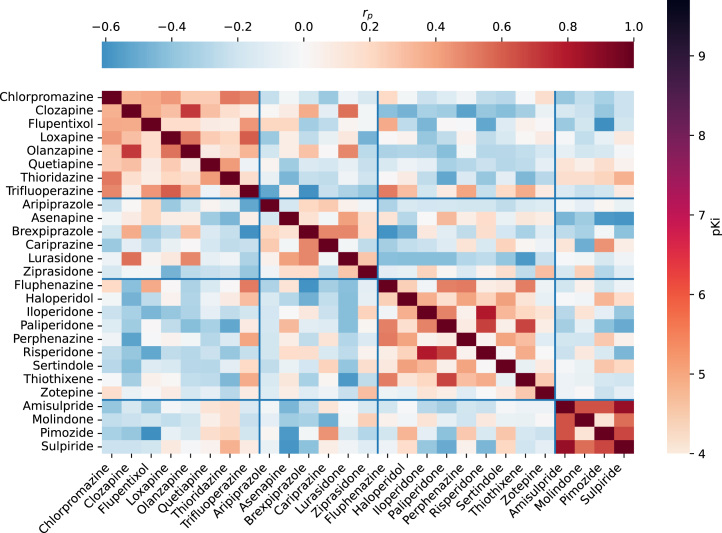
Antipsychotic clustering based on receptor profiles. The color of each small square indicates the strength of correlation between the receptor profile of the antipsychotic in the corresponding row and column (e.g., one can see that pimozide shows a similar receptor profile to amisulpride but not to flupentixol). The grouping outlined by the blue lines reflects the result of a clustering algorithm that aims to group highly correlated drugs together.

Antipsychotics were clustered based on the similarity of their receptor affinity profiles. Four clusters were identified (Figure 2).

### Characterizing Cluster Receptor Profiles

**Figure 3:**
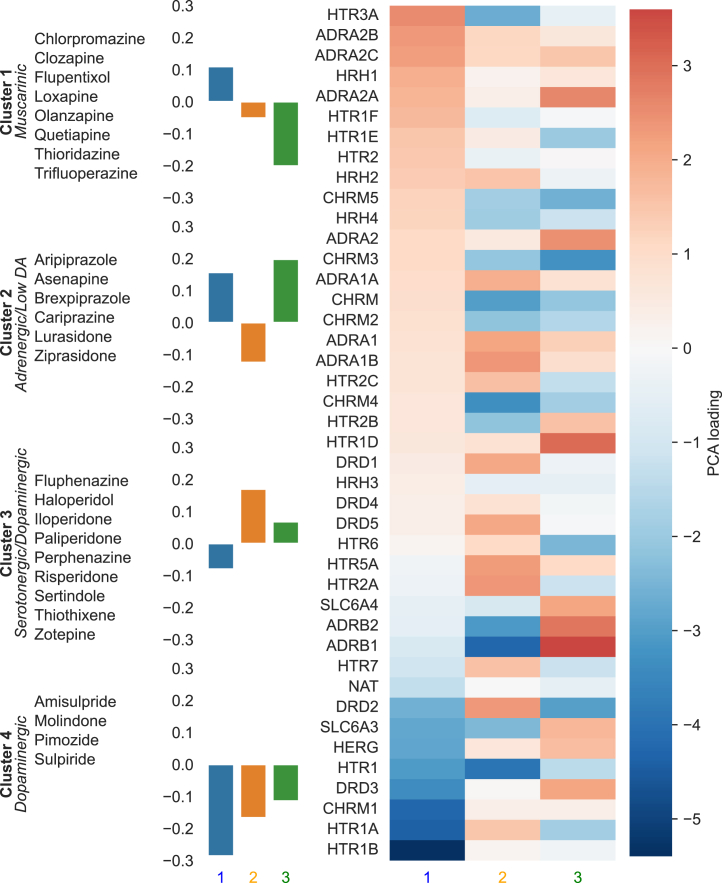
Characterizing receptor-defined antipsychotic clusters. The numbers 1, 2, and 3 refer to the first 3 principal components. The bar chart shows that, e.g., cluster 4 has a large negative loading for component 1. The heatmap shows how the components relate to the receptor profile. The large negative loading for component 1 in cluster 4 indicates that the drugs in this cluster will tend to act as relatively strong antagonists at HTR1 and CHRM1 and weak antagonists (or even agonists) at ADRA2B and ADRA2C. DA, dopamine; PCA, principal component analysis; QTc, corrected QT interval.

To summarize the receptor affinity profile of each cluster, we examined the mean loading for the 3 PPCA components explaining the greatest variance (Figure 3). These 3 components explained 65% of the variance, with the next largest component contributing 9%. The first cluster (chlorpromazine, clozapine, flupenthixol, loxapine, olanzapine, quetiapine, thiordiazine, and trifluoperazine) was characterized as muscarinic given its strong negative loading on the third component, which reflected antagonism at the muscarinic M_2_–M_5_ receptors (but either agonism or weak antagonism at the M_1_ receptor). The second cluster (aripiprazole, asenapine, brexpiprazole, cariprazine, lurasidone, and ziprasidone) was characterized as adrenergic with low dopaminergic antagonism and had a strong positive loading on the third component and a negative loading on the second component, reflecting a lack of muscarinic or serotonergic antagonism but significant adrenergic antagonism and dopamine D_2_ partial agonism. The third cluster (fluphenazine, haloperidol, iloperidone, paliperidone, perphenazine, risperidone, sertindole, thiothixene, and zotepine) was characterized as serotonergic-dopaminergic due to its strong positive loading on the second component, which reflects serotonergic and dopaminergic antagonism. The fourth cluster (amisulpride, molindone, pimozide, and sulpiride) was characterized as dopaminergic given its strong negative loading on the first component, which reflects relatively pure dopaminergic antagonism without adrenergic effects.

### Clinical Profiles of Receptor-Defined Taxonomies

**Figure 4:**
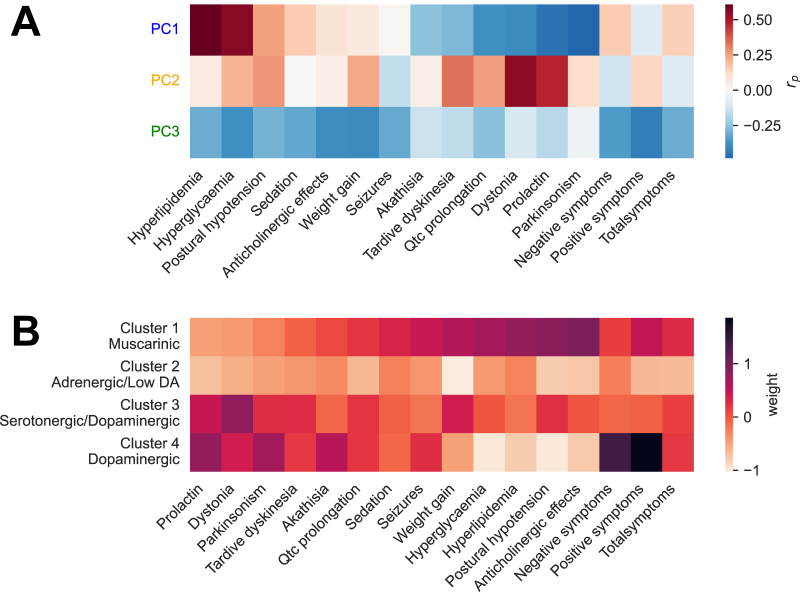
Characterizing clinical profiles of principal components (PCs) and receptor-defined clusters. **(A)** Correlation coefficients across antipsychotics between principal component loadings illustrated in Figure 3 and their clinical effects. Red indicates that a drug with a strong positive loading for that component is likely to be associated with the effect in question. **(B)** Mean scores for antipsychotic clusters illustrated in Figure 2, a darker color indicates that the cluster is associated with greater severity of the side effect (or greater efficacy for symptom measures) in question. DA, dopamine.

We then characterized how these principal components of receptor affinity correlated with clinical effects (Figure 4A). Drugs with a positive loading for the first principal component are more likely to cause metabolic and cholinergic side effects but have a low propensity for Parkinsonism, akathisia, and hyperprolactinemia; they also show the greatest efficacy for total symptoms. Drugs with a positive loading for the second component show the opposite pattern, with a relative propensity to cause Parkinsonism, akathisia, and hyperprolactinemia over metabolic effects; they also show the greatest efficacy for positive symptoms. A positive loading for the third component reflects a propensity for a generally low all-round side-effect burden and also less efficacy in terms of total, positive, and negative symptoms.

We also looked at the mean clinical effect scores for the receptor-defined clusters (Figure 4B). We found that cluster 1 was associated with anticholinergic side effects, postural hypotension, and metabolic side effects; cluster 2 was associated with a globally low side-effect burden; cluster 3 was associated with a globally moderate side-effect burden; and cluster 4 was associated with Parkinsonism, akathisia, and hyperprolactinemia. Clusters 1 and 4 were more efficacious than clusters 2 and 3.

**Figure 5:**
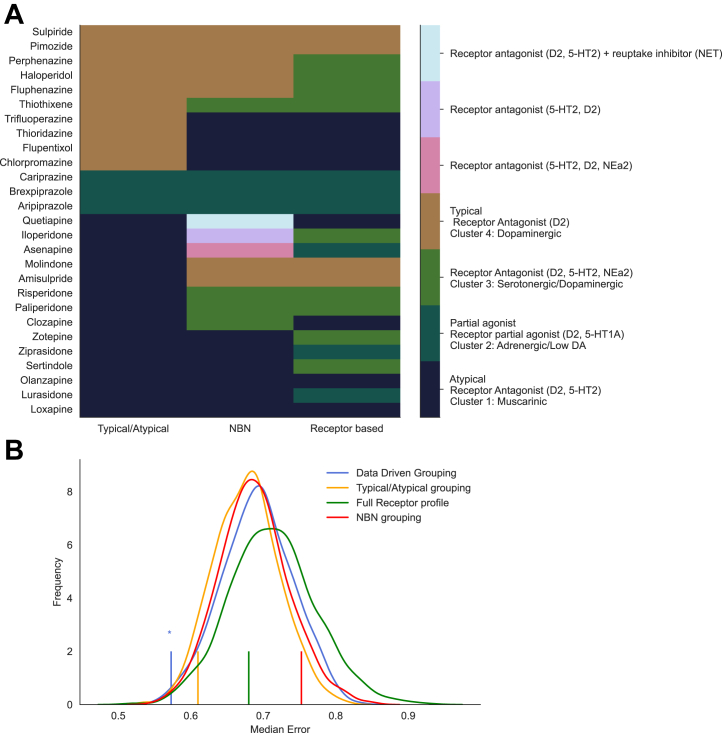
Antipsychotic categorization schemes and prediction of clinical effects. **(A)** Antipsychotics classified according to a typical/atypical/partial agonist split, neuroscience-based nomenclature (NBN), and the receptor-defined clusters illustrated in Figure 2. **(B)** The curves illustrate the permutation-generated null distribution. Vertical lines indicate the observed median error for predicting out-of-sample clinical effect profiles (a smaller value reflects a more accurate prediction). The data-driven and typical/atypical groupings produce a statistically significant prediction of overall clinical profile compared with the null distribution.

Finally, we examined which of the 4 classification methods (the receptor profile–defined grouping described here, complete receptor binding profiles, NbN-defined grouping, and atypical/typical/partial agonist-defined groupings) (Figure 5A) could best predict out-of-sample clinical effect profiles (Figure 5B). Only the receptor-defined clusters described in this article produced a statistically significant prediction (*p* = .008), in contrast to the typical/atypical/partial agonist (*p* = .06), complete receptor profile (*p* = .30), or NbN (*p* = .90) groupings.

## Discussion

This article illustrates how receptor profiles can be used to classify antipsychotics in a data-driven fashion. Furthermore, we demonstrate that the groupings derived from this approach can predict clinical effect profiles.

These findings have several implications. An unbiased pharmacologically driven approach to classification has a priori advantages in that, by definition, it reflects pharmacology and does not require decisions regarding which receptors to prioritize. In addition, we have demonstrated how receptor profiles can be used to quantitatively estimate clinical effects, which has potential uses when evaluating compounds that have not yet undergone clinical testing. Furthermore, treatment side effects are key factors that people with schizophrenia and their clinicians consider when making prescription decisions (24), and reducing side effects of antipsychotics is central to initiatives to improve morbidity and mortality rates in this patient group (25). Although treatment decisions based on the side-effect burden may be best made at the individual drug level, we have identified groups of antipsychotics with similar receptor binding signatures and either globally low or moderate side-effect burdens; this has the potential to inform clinical practice. For example, we identified a group of antipsychotics with a low side-effect risk that included all licensed partial agonists alongside ziprasidone and its structural analog lurasidone. Previous studies and clinical guidance documents have recommended that this same group of antipsychotics be selected preferentially when there is a desire to avoid metabolic side effects (5,16); this is consistent with our data-informed classification scheme, but our scheme extends this, indicating that they are preferential in terms of other side effects as well. Thus, guidelines and clinicians may recommend a drug from this group as the first-line treatment, given the overall favorable side-effect profile and as a rational choice to switch to for patients experiencing metabolic side effects from a drug not in this class. In contrast, if efficacy is paramount, then it may be preferable to consider clusters 1 or 4, with the decision between these dependent on whether hyperprolactinemia/movement or metabolic side effects are a greater concern.

In terms of treatment effectiveness, it is unclear how to select a second antipsychotic in the case of initial nonresponse. There is evidence that switching to a pharmacologically distinct compound produces clinical benefits (13). Although current guidelines do recommend switching to a different antipsychotic class prior to considering clozapine, this guidance is often limited to switching between atypical/typical agents (14). The current classification separates drugs into classes as pharmacologically distinct from one another as possible, potentially providing some guidance as to sensible switching choices when changing medication secondary to lack of effectiveness. While antipsychotic polypharmacy should typically be avoided, the classification could also be of use in suggesting more effective antipsychotic combination strategies in cases in which other options have been exhausted (26,27). Further work definitively testing whether these groupings reflect an optimal switching strategy is warranted.

Despite these advantages, alternative taxonomies offer benefits in other aspects. For example, the NbN approach encompasses psychopharmacological treatments as a whole, as opposed to antipsychotics only. Future work could similarly extend the current approach to a wider range of compounds. A drawback of the NbN approach is the necessary limitation to covering an incomplete range of receptor systems. While this has the benefit of keeping the number of systems manageable for the user, it means that important facets are neglected. For example, histaminergic and muscarinic affinities do not feature in the NbN approach to classifying antipsychotics (28). This is a significant limitation given the central role of the histaminergic system in determining the propensity of a drug to induce weight gain and sedation (28,29) and the fact that muscarinic mechanisms underlie the efficacy of several antipsychotics currently in phase 3 trials (30,31). The fact that histaminergic affinities contribute to the clustering approach used in the current analysis may therefore be one of the factors that improve its ability to predict clinical effect profiles compared with NbN. There is not, however, a clean delineation between receptor types and the data-driven clusters. For example, medications in both clusters 1 (e.g., olanzapine, quetiapine, and clozapine) and 3 (zotepine and thiothixene) show relatively high levels of H_1_ receptor affinities. In many situations, both clinically and in research, it will be preferable to consider compounds individually and select a drug based on its unique clinical or receptor profile as opposed to its membership of any grouping. In some respects, the present findings do not necessarily identify an optimal clustering but highlight the shortcomings of existing taxonomies.

In the current analysis, we attempt to move away from preexisting biases by using an agnostic classification algorithm. However, to obtain more fully unbiased results, one requires unbiased data in addition to an unbiased algorithm. The database chosen does not reflect an entirely systematic survey of receptor affinities but, to an extent, reflects research interests. This means that for some drugs, such as lurasidone, flupentixol, and sulpiride, there is a paucity of data, although for the rest, the database is relatively comprehensive. However, while this is a potential limitation of our approach, it is even more so for the NbN approach because it uses a subset of known receptor affinities. Moreover, it is partially mitigated by the fact that drugs typically undergo standard receptor affinity screening. There are also potential biases in the clinical data used in the partial least squares analysis. The effect sizes are obtained from clinical trials that in many cases used significantly higher doses in trials of typical compared with atypical drugs. This means that in some cases, the side effect magnitude may reflect these dosing practices in addition to the pharmacodynamic properties of the drug. This will, in turn, lead to a potential overestimation of the ability of the typical/atypical grouping to predict side effects (32,33).

Our approach can be readily updated with new findings as they emerge, and we have made the required code openly available. However, the optimal cluster solution may change with the addition of drugs, particularly when considering medications with entirely novel mechanisms of action (30,34). Similarly, for drugs currently missing significant amounts of data, the addition of data may change the assigned cluster or the entire cluster solution. The relatively small sample size of antipsychotics with available clinical data means that not only the cluster solution may be subject to subsequent change but also that its prediction of effects should not be taken as clear evidence of superiority. It may often be preferable to consider compounds as unique rather than as part of any grouping.

One of the main areas in which our methods could potentially be improved is the accounting for additional pharmacological properties. Our approach focuses on relative receptor affinities; therefore, while this adjusts for dosing differences, it does not consider that active metabolites may have quite different pharmacodynamic effects than the parent compound. The fact that functional assays were not used also means that the degree of partial agonism could not be quantified, and only a binary categorization was used. Some receptors that may have clinically relevant effects did not have sufficient data available to be included in the analysis. Additionally, all binding was quantified using in vitro assays and in vivo values may differ. The fact that compounds differ in their ability to cross the blood-brain barrier is also not accounted for, which means that the relationship between lower permeability and greater peripherally mediated side effects will not be reflected. Finally, in the cases in which individual receptors mediate the majority of an effect (e.g., human ether-a-go-go-related gene and corrected QT interval prolongation), it may be that the importance of the receptor becomes lost in the analysis. Our examination of the association between taxonomies and clinical effects was solely based on data obtained from trials in individuals with schizophrenia. Antipsychotics are, however, also used in other disorders, and, at least in terms of efficacy, they demonstrate interdrug differences distinct from those observed in trials of psychotic disorders (6,35,36).

Future work may consider a data-driven approach to clustering all neuropsychiatric medication, as opposed to medications used solely in the treatment of psychosis. To build upon the current approach, clinical studies could evaluate the benefits of the classification scheme in guiding switching decisions, while the predictive model may be of use in identifying ideal receptor profiles that maximize efficacy while minimizing side effects. Additional analyses could also attempt to identify whether certain receptor profiles display disorder-specific efficacy.

In conclusion, this study provides a pharmacological data-driven approach to the classification of antipsychotic medication. We derived 4 groups of antipsychotics with distinct receptor, efficacy, and side-effect profiles. This approach reflects the pharmacological properties as closely as possible. It also shows considerable mapping to clinical effect profiles, suggesting that it may hold some advantages over existing approaches. This data-driven taxonomy promises to benefit both patients and researchers, guiding appropriate treatment and drug development in the future.

## Supplementary Material

Supplementary Material

Key Resources Table
