# Supplementary material for: Data-Driven Taxonomy for Antipsychotic Medication: A New Classification System
Source: Biol Psychiatry. 2023 Oct 1;94(7):561–8. doi: 10.1016/j.biopsych.2023.04.004 (PMC10914668; doi:10.1016/j.biopsych.2023.04.004)
Supplement: Supplementary Material [file mmc1.pdf]

SUPPLEMENTARY INFORMATION

Data-driven Taxonomy for Antipsychotic Medication: A New Classification System

McCutcheon *et al.*

Supplemental Methods

Receptor Data
Levopromazine and penfluridol had insufficient receptor data to be included:

The following receptors had insufficient drug data to be included:
Adenosine receptors 1, 2A, and 3
Adrenoceptors Alpha1D and Beta 3
Cholecystokinin B
Cholinergic Receptor nicotinic alpha 4 subunit
Cannabinoid Receptor 1
Cannabinoid Receptor 2
Delta1 Opioid Receptor
Tachykinin Receptors 1, 2, and 3

The following receptors' drug data did not differ between drugs:
Cholinergic Receptor Nicotinic subunits A1B2, A2B2, A2B4, A3B2, and A7
Mu and Kappa Opioid Receptors
Prostaglandin E Receptor 3
Substance P receptor
Thromboxane A2 receptor
Vasopressin 3 receptor

Clustering
Prior to probabilistic PCA (PPCA) pKi values were standardised by subtraction of the mean and scaling to unit variance. PPCA is similar to principal components analysis (PCA) in that it is a dimensionality reduction technique that identifies a lower dimensional latent space. Unlike PCA, PPCA has an associated probabilistic model for the observed data, this has the advantage that it can be fit to data where some values are missing. The probabilistic model explicitly includes a noise term in the generative model and when model fitting.

PPCA imputes missing data points by first estimating the distribution of compressed information based on non-missing data, and then reconstructing missing data from the

compressed information as estimated data points. This process is iteratively performed using an Expectation–Maximization (EM) algorithm.

The EM algorithm iteratively calculates the maximum likelihood estimate of the incomplete data set. Each iteration of the EM algorithm consists first of an 'expectation' step in which in which the missing values are imputed as described above, the maximization step then updates the imputed parameters using the complete dataset constructed in the previous expectation step. These steps are repeated until there is no further improvement in likelihood estimate.[1,2]

The Louvain clustering algorithm was used to group antipsychotics with similar receptor profiles. The gamma parameter was set at '1', negative and positive correlations were equally weighted, and the algorithm was ran 100 times with the solution showing the greatest modularity chosen.

Side effects data

We performed a systematic 'umbrella' review of meta-analyses to create a database of effect size magnitudes for antipsychotic side effects. As part of another ongoing project we simultaneously searched for antidepressant-associated effects. We searched Pubmed from inception to August week 4 2022. We focused on key side effects described in The Maudsley Prescribing Guidelines in Psychiatry.[3] As such, the following search terms were used: [(antidepressant OR antipsychotic) AND (extrapyramidal OR parkinsonism OR dyskinesia OR dystonia OR akathisia OR prolactin OR headache OR agitation OR insomnia OR cholinergic OR gastrointestinal OR constipation OR nausea OR arrythmia OR QTc OR hypotension OR hypertension OR weight OR glucose OR lipid OR cholesterol OR triglyceride OR sedation OR natraemia OR natremia OR sodium OR thromboembolism OR sexual) AND "meta-analysis"]. Only meta-analyses based on data from randomised controlled trials were considered. Where there was more than one meta-analysis for a given side effect, the study with the largest sample size was selected. For each side effect and medication, we extracted effect size magnitude compared to placebo. Where fewer than 2/3 of antipsychotics had meta-analytic side effect estimates available, we aimed to use more comprehensive side effect data sources in the form of ordinal rankings from national/international guidelines or consensus statements for treatment of schizophrenia.[3–10] These guidelines were selected in line with previous meta-reviews in the field.[11] The resultant side effect database consisted of rows corresponding to drugs, and columns corresponding to side effects, with entries corresponding to either meta-analysis derived effect sizes or guideline-defined ordinal scores. Each side effect was then normalised (minimum-maximum scaled by subtracting the minimum value for that side effect and dividing by the range) to give values between 0 and 1.

Of 2060 citations retrieved, 11 meta-analyses met inclusion criteria. For antipsychotics, side effect data were available for 32 drugs. Six side effects (weight gain, Parkinsonism, akathisia, anticholinergic effects, sedation, and hyperprolactinaemia) had meta-analytic data available for ≥66% of available drugs (≥22 drugs); these data were extracted from 2 NMAs.[12,13] Antipsychotic side effect data for remaining side effects were derived from national/international guidelines or consensus statements. Up to 4 guidelines/consensus statements[3,8–10] provided ordinal rankings of 7 antipsychotic-related side effects for which

sufficient meta-analytic data were not available (namely: QT prolongation, orthostatic hypotension, dystonia, tardive dyskinesia, seizure, dyslipidaemia, and dysglycaemia). Ordinal scores for drug side effects were extracted from each guideline and normalised (minimum-maximum scaled by subtracting the minimum value for that side effect and dividing by the range); where there were multiple guidelines, a mean of normalised scores was calculated. Missing side effect values were imputed using probabilistic PCA

Partial Least Squares
Side effect scores were highly skewed, therefore scores were shifted to have a zero floor before undergoing a square root transform and standard scaling (removal of mean and scaling to unit variance). Scaling was fit only on training data, before later being applied to left out test sample. Partial least squares was implemented using the nipals algorithm with 3 components.

Supplemental References

1. Hegde, H. *et al.* MICE vs PPCA: Missing data imputation in healthcare. *Informatics in Medicine Unlocked* **17**, 100275 (2019).
2. Tipping, M. E. & Bishop, C. M. Probabilistic principal component analysis. *J. R. Stat. Soc. Series B Stat. Methodol.* **61**, 611–622 (1999).
3. Taylor, D. M., Barnes, T. R. E. & Young, A. H. *The Maudsley Prescribing Guidelines in Psychiatry*. (Wiley Blackwell, 2021).
4. NICE. in *Psychosis and schizophrenia in adults: prevention and management* (2014).
5. Barnes, T. R. E. & Psychopharmacology, British Assoc. Evidence-based guidelines for the pharmacological treatment of schizophrenia: recommendations from the British Association for Psychopharmacology. *Journal of Psychopharmacology* **25**, 567–620 (2011).
6. IPAP. International Psychopharmacology Algorithm Project (www.ipap.org): Schizophrenia Algorithm Flowchart. (2019).
7. Verma, S. *et al.* Ministry of Health Clinical Practice Guidelines: Schizophrenia. *Singapore Medical Journal* **52**, 521–525 (2011).
8. McGorry, P. *et al.* Royal Australian and New Zealand College of Psychiatrists clinical practice guidelines for the treatment of schizophrenia and related disorders. *Australian and New Zealand Journal of Psychiatry* **39**, 1–30 (2005).
9. Falkai, P. *et al.* World Federation of Societies of Biological Psychiatry (WFSBP) - Guidelines for biological treatment of schizophrenia, part 1: Acute treatment of schizophrenia. *World Journal of Biological Psychiatry* **6**, 132–191 (2005).
10. APA. The American Psychiatric Association Practice Guideline for the Treatment of Patients With Schizophrenia. Third Edition. (2020).
11. Howes, O. D., Thase, M. E. & Pillinger, T. Treatment resistance in psychiatry: state of the art and new directions. *Mol Psychiatry* **27**, 58–72 (2022).
12. Huhn, M. *et al.* Comparative efficacy and tolerability of 32 oral antipsychotics for the acute treatment of adults with multi-episode schizophrenia: a systematic review and network meta-analysis. *Lancet* **394**, 939–951 (2019).
13. Zhu, Y. *et al.* Prolactin levels influenced by antipsychotic drugs in schizophrenia: A systematic review and network meta-analysis. *Schizophr. Res.* **237**, 20–25 (2021).
